# Enhanced Oxidative Stability and Bioaccessibility of Sour Cherry Kernel Byproducts Encapsulated by Complex Coacervates with Different Wall Matrixes by Spray- and Freeze-Drying

**DOI:** 10.1021/acsomega.3c02128

**Published:** 2023-06-23

**Authors:** Umit Altuntas, Gokce Altin-Yavuzarslan, Beraat Ozçelik

**Affiliations:** † Food Engineering Department, Chemical and Metallurgical Engineering Faculty, Istanbul Technical University, 34469 Istanbul, Türkiye; ‡ Food Engineering Department, Faculty of Engineering and Natural Sciences, Gümüşhane University, 29100 Gümüşhane, Türkiye; § Molecular Engineering & Sciences Institute, University of Washington, 3946 W Stevens Way NE, Seattle, Washington 98105, United States; ∥ BIOACTIVE Research and Innovation Food Manufac. Indust. Trade Ltd., Teknokent ARI-3, B110, 34467 Istanbul, Turkey

## Abstract

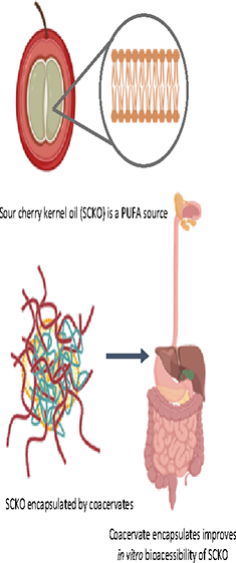

Sour cherry (*Prunus cerasus* L.) seeds are obtained as byproducts of the processing of sour cherries into processed foods. Sour cherry kernel oil (SCKO) contains n-3 PUFAs, which may provide an alternative to marine food products. In this study, SCKO was encapsulated by complex coacervates, and the characterization and in vitro bioaccessibility of encapsulated SCKO were investigated. Complex coacervates were prepared by whey protein concentrate (WPC) in combination with two different wall materials, maltodextrin (MD) and trehalose (TH). Gum Arabic (GA) was added to the final coacervate formulations to maintain droplet stability in the liquid phase. The oxidative stability of encapsulated SCKO was improved by drying on complex coacervate dispersions via freeze-drying and spray-drying. The optimum encapsulation efficiency (EE) was obtained for the sample 1% SCKO encapsulated with 3:1 MD/WPC ratio, followed by the 3:1 TH/WPC mixture containing 2% oil, while the sample with 4:1 TH/WPC containing 2% oil had the lowest EE. In comparison with freeze-dried coacervates containing 1% SCKO, spray-dried ones demonstrated higher EE and improved oxidative stability. It was also shown that TH could be a good alternative to MD when preparing complex coacervates with polysaccharide/protein networks.

## 1 Introduction

In 2011, the Food and Agriculture Organization (FAO) reported that fruit and vegetable byproducts account for the majority of food losses and waste.^1^ However, fruit and vegetable wastes are also rich in micronutrients and phytochemicals.^2^ For this reason, these kinds of byproducts can be considered as sustainable sources of bioactive compounds for the food, cosmetic, and pharmaceutical industries. Previously, the functioning of cocoa shell waste^3^ and black radish husk waste^4^ were investigated. Encapsulation of valuable oil from sour cherry kernel byproducts and thus improving the oxidative stability and in vitro bioaccessibility of fatty acids of oil by encapsulation using complex coacervation is a notable issue. Sour cherries (*Prunus cerasus* L.) are grown throughout the world at a rate of 1.2 million tons per year.^1^ In the food industry, sour cherries are commonly used for juice production and mostly consumed as processed products, including canned and frozen sour cherries, sour cherry juice,^5^ nectars, soft drinks and alcoholic beverages, jams, and food manufacturing additives.^6,7^ However, when sour cherries are processed into processed foods, large amounts of seeds are discarded. The byproducts of sour cherry processing, especially sour cherry kernel (SCK), could be used as a dietary fiber, protein, and fat source.^7^ SCKs contain 32–36% oil that is rich in unsaturated fatty acids and beneficial compounds including tocopherol and β-sitosterol. Sour cherry kernel oil (SCKO) is high in oleic acid (50–53%) and linoleic acid (roughly 35–38%). SCK has a significantly high oil content (17.0%) compared to other oilseeds and tree fruits, such as soybean (18–21%), corn (3–6%), sunflower (36–44%), and olive (16–36%), making it a useful source of edible oil.^8^ A major component of SCK oil is linoleic and linolenic acids, classified as −6 and −3 PUFAs, respectively, and oleic acid, classified as MUFA. The proportion of linoleic and linolenic acids is over 45%, while the total SFAs (mainly stearic and palmitic acids) are less than 10%.^9^ In addition, SCK oil contains phenolic compounds as well as tocopherols and beta-carotene.^7,10^ Therefore, SCKO gains special interest as a potential candidate bioactive ingredient.

It is well known that biologically active compounds are sensitive to unfavorable environmental conditions like oxygen, light, pH changes, or moisture. At this point, encapsulation technology provides a suitable platform to protect them from these conditions and enhance their bioactive stability during storage, in food formulation, and in the gastrointestinal tract after consumption.^10,11^ Among the encapsulation techniques, coacervation is one of the suitable approaches for the encapsulation of lipophilic compounds. In this technique, the lipophilic bioactive compound is introduced into an emulsion system. Then, phase separation is maintained by using two or more biopolymers applied to suspended solids or the emulsion (core material) to form a sealing membrane layer.^12^

Proteins belong to the amphiphilic polymers, which have both hydrophilic and hydrophobic portions. Therefore, they can adsorb strongly at the oil–water interface through electrostatic and/or steric repulsion.^13^ Since the techno-functional properties of proteins are affected by various parameters such as pH, temperature, organic solvents and ionic strength, their industrial use as a wall material in the encapsulation process is limited.^14^ At this point, polysaccharides offer an alternative as a biopolymeric wall material that is more stable to environmental conditions than polymers. However, they alter the rheology of the dispersed phase.^15^ For this reason, the use of a protein/polysaccharide combination in the wall matrix has been proposed.^16,17^ Recently, it has been shown that the use of the combination of whey protein concentrate, maltodextrin, and gum Arabic as wall materials for the encapsulation of krill oil leads to desirable oxidative stability during storage as well as in the simulated gastrointestinal environment.^18^ Whey protein concentrate (WPC) is widely used as a protein ingredient in the food industry due to its nutritional qualities and special mechanical properties such as gelation and emulsion stabilization.^16^ Gum Arabic (GA) is a compound edible polysaccharide^16^ that is commonly used as a stabilizer in the food industry.^19^ While the multipolymeric wall matrix increases the stability of the encapsulated bioactive compounds, the encapsulated substances are still not thermally stable in the liquid phase.^20^

Spray and freeze-drying are the two common methods used in the food industry to obtain solid phase encapsulations. In the past, flaxseed oil and shea oil emulsions were converted into solid forms by freeze-drying.^21,22^ In the drying process, the presence of a carrier in the solution improves the efficiency of the drying process and protects the encapsulations from thermal deterioration by acting as an additional protective layer.^3^ MD and TH are among the commonly used materials for this proposal, especially in spray-drying.^23,24^ It also has some additional advantages such as mild taste, low cost, and low viscosity.^16,17,25^ The aim of this study is to use one of the food wastes, SCK, as a source of lipophilic bioactive compounds (oil fraction) and to improve the kinetic and thermal stability as well as the in vitro bioaccessibility (%) of these encapsulations by encapsulating this unique oil by the complex coacervation method followed by spray and freeze-drying. We hypothesize that TH can be a good alternative to MD in the preparation of complex coacervates with polysaccharide and protein networks, and it can be processed in spray and freeze-drying. The effects of the composition of the multiwalled material, the concentration of the SCKO, and the drying methods on the encapsulation efficiency (EE), oxidative stability, and in vitro bioaccessibility (release %) of the SCKO were evaluated in terms of the physical characterization of the encapsulations and the chemical stability of the encapsulated kernel oil. This is, to the best of our knowledge, the first study on extraction of SCKO from the sour cherry processing byproduct to be used as a bioactive food ingredient.

## 2 Materials and Methods

### 2.1 Materials

SCKs were obtained from local suppliers and extracted by the classical solvent extraction method (using n-hexane as a solvent and the soxhlet method for a 5 h extraction process) to obtain SCKO. The following ingredients were bought from Aromsa Co. (Kocaeli, Turkey): maltodextrin DE 20 (MD), trehalose (TH), GA, and WPC. Besides, pepsin, pancreatin, porcine bile extract, thiobarbituric acid, and other chemicals were of analytical grade and purchased from Fluka Co. All chemicals were of pure grade.

### 2.2 Encapsulation of SCKO by the Complex Coacervation Method

Table 1 shows the different formulations prepared to produce the wall matrix of the encapsulations. The wall matrix of the coacervates was prepared using different biopolymers and preparation techniques as reported by El-Messery et al.^18^

**Table 1:** Formulations of Complex Coacervates in Terms of Rate of Wall Materials in the Solution (Maltodextrin, MD; Whey Protein Concentrate, WPC and Trehalose, TH) and the Amount of SCKO Found in the Solution

sample code	MD:WPC ratio (by weight)^a^	SCKO conc. (%) (in total weight)^a^
MD 1	4:1	1
MD 2	3:1	1
MD 3	4:1	2
MD 4	3:1	2
	**TH:WPC ratio**	**SCKO conc. (%)**
TH 1	4:1	1
TH 2	3:1	1
TH 3	4:1	2
TH 4	3:1	2

a GA (1% (w/v)) was added into the final formulations to maintain coacervate stability in the liquid phase.

For this purpose, the biopolymers were first mixed with water. MD (10% w/v), TH (10% w/v), and GA (10% w/v) were mixed with distilled water at 50–60 °C and dissolved with stirring for 1 h. WPC was previously dissolved in distilled water at 60–80 °C for 30 min before being added to the formulation. Then, the solutions of the wall material were mixed with two different percentages (1 and 2% w/v) of SCKO and homogenized using an Ultra-Turrax homogenizer at 18,000 rpm for 5 min. To obtain coacervates of uniform and small size (in nanometers), each formulation was passed five times through a microfluidizer at 25.000 psi homogenization pressure. Finally, the solution was added to GA and each formulation was homogenized for 5 min using the Ultra-Turrax homogenizer to maintain the kinetic stability of the coacervates in the liquid phase.

### 2.3 Solidification of Coacervate Dispersions by Spray-Drying and Freeze-Drying

**Figure 1:**
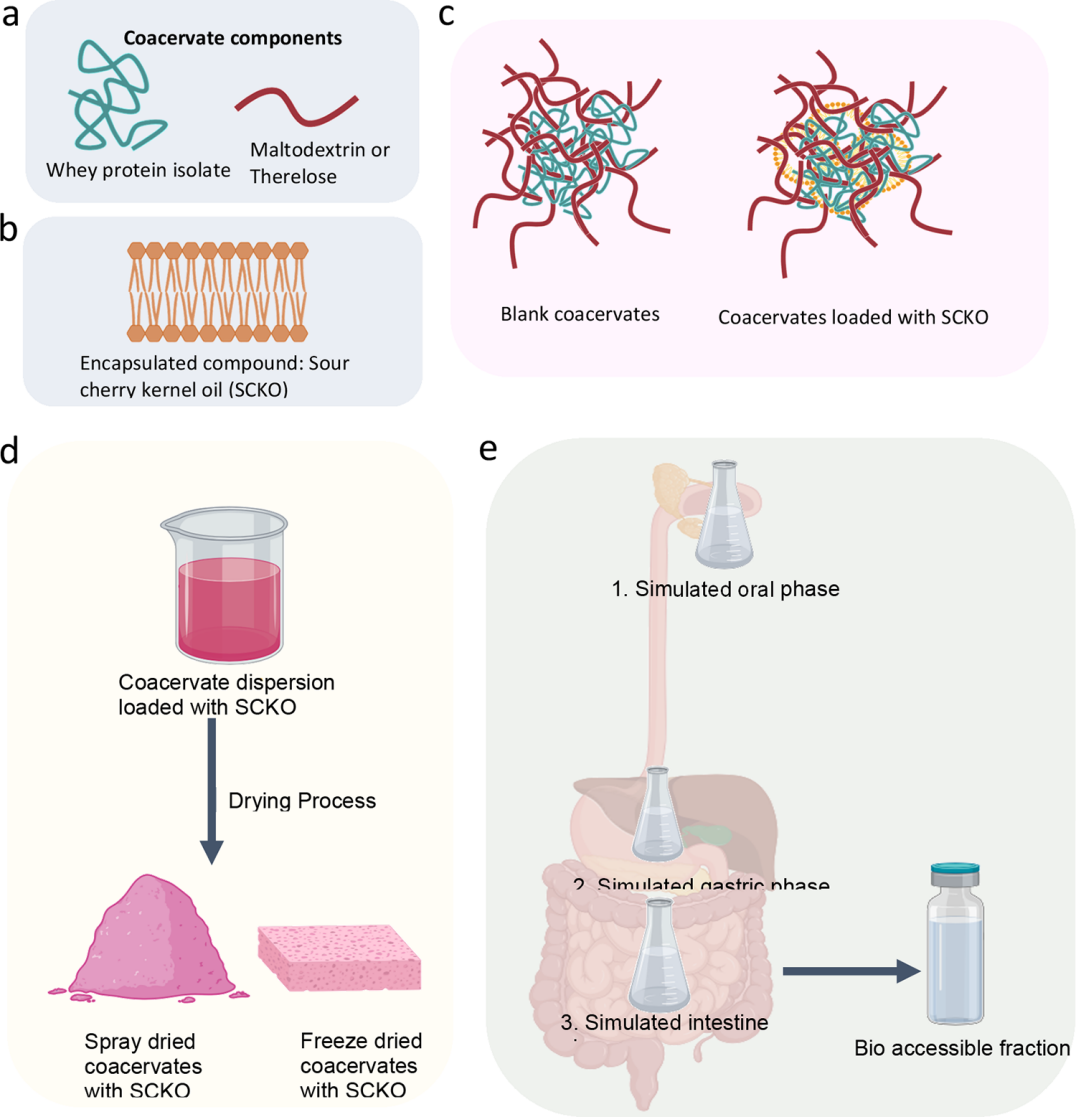
Schematic representation of the experimental section. Components of coacervates (a, b); Encapsulated bioactive compound, SCKO (b); Blank and SCKO loaded coacervates (d), Drying of coacervate dispersion with SCKO by using spray-drying and freeze-drying (e); In vitro bioaccessibility of SCKO in spray-dried and freeze-dried coacervate dispersions.

The coacervate containing formulations were divided into two portions to obtain the coacervates in different solid phases. The first portion was dried using a laboratory-scale spray dryer (Mini spray dryer B-290, BÜCHI Labortechnik, Switzerland). The emulsion was pumped into the dryer using a peristaltic pump with a flow rate of 5 cm^3^/min. The flow rate and pressure of the drying air were set at 2.5 m^3^/min and 0.06 MPa, respectively. The inlet temperature was 130 °C and the outlet temperature was 71 °C. The powdered microcapsules were collected and stored in an airtight desiccator for subsequent analysis. The second portion was frozen overnight at −20 °C and then freeze-dried using a freeze dryer (Christ Alpha 1-2D plus, Germany). The temperature of the ice condenser was adjusted to −50 °C, and the vacuum pressure was set at 0.04 mbar. After the frozen samples were dried for 48 h, the dried coacervate was collected, crushed, and stored in an airtight desiccator for subsequent testing. A schematic representation of the experimental section is given in Figure 1.

### 2.4 Characterization of the Coacervate Dispersions

#### 2.4.1 Determination of the Physical Stability of Coacervate Dispersions

The separation of the serum within the liquid phase was taken as an indication of the stability of the coacervate. The coacervate solutions were transferred to a 20 mL cylinder, sealed, and stored at 25 °C for five different time periods (24, 48, 72, 96, and 120 h). Equation 1, adapted from El-Messery et al.,^18^ was used to calculate the percent separation of serum from the coacervate solution based on the amount of serum separated from the coacervate solution.
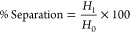
1where *H*_1_ is the height of the upper phase and *H*_0_ is the starting height.

#### 2.4.2 Measurement of the Particle Size and ζ-Potential

The mean particle size and ζ-potential of the coacervates in the liquid phase were measured according to El-Messery et al.^18^ by using a Zetasizer (Malvern Inst, Worcestershire, UK). Just before the measurements, samples were diluted 100-fold using distilled water, thoroughly stirred, and placed in a quartz cuvette to minimize the effects of multiple scattering. The diluted samples containing coacervates were loaded into a foldable capillary electrophoresis cell at a count rate between 100 and 300 Kcps to perform measurements in triplicate.

#### 2.4.3 Determination of the Oxidative Stability of Encapsulated SCKO in Storage

##### 2.4.3.1 Measurement of the Peroxide Value (PV)

The dried coacervate dispersions were placed in 50 mL disposable polypropylene centrifuge tubes and incubated at 55 ° C in the dark for 15 days. Samples were taken at 2-day intervals. The lipid hydroperoxides were measured by using the method of Shantha and Decker.^26^ Lipids were extracted from the sample (0.3 mL) by adding 1.5 mL of 2-propanol /isoctane mixture (at 1:3 ratio, v/v) and shaking for 10 s, after centrifuging three times for 2 min. The top layer was aliquoted (0.2 mL) and mixed with 2.8 mL of methanol/1-butanol mixture (2:1 v/v), followed by the addition of 30 μL of a mixture of iron(II) solution/ammonium thiocyanate (a 1:1 (v/v), 3.94 M). After 20 min, a UV–visible spectrophotometer was used to measure absorbance at 510 nm.

##### 2.4.3.2 Measurement of TBARS (Thiobarbituric Acid-Reactive Substances)

The dried coacervate dispersions were incubated at 55 °C for 15 days and samples were taken every 2 days for assays. The method of McDonald and Hultin^27^ was used to measure TBARS. The sample (1 mL) and 2 mL of the TBA reagent were placed in a screw-capped glass tube and then heated in a water bath (90 °C) for 15 min. The tubes were then placed in a water bath at 24 °C to cool for 10 min. The tubes were centrifuged at 10,000 rpm for 15 min and then again for 10 min. The absorbance of the supernatant was measured at 532 nm. TBARS concentration was quantified in nanomolar units (nM) using a standard curve of 1,1,3,3-tetraethoxypropane at values between 0 and 20 nM. All samples were measured in triplicate.

### 2.5 Determination of the EE of Dried Coacervates

The encapsulation efficiency (EE) of the dried coacervates was calculated according to the method of El-Messery et al.^18^ Fifteen milliliters of hexane and 1.5 g of the sample were placed in a glass jar and shaken for 2 min to extract unencapsulated (free) oil from the coacervates in the powder form. The extract was filtered three times through a No.1 Whatman filter paper, and the powder collected on the filter was rinsed three times with 20 mL of hexane to increase the efficiency of the extraction. At 60 °C, the combined extracts were evaporated to dryness until the weight was constant, corresponding to the unencapsulated oil.

EE was calculated using the following equation:
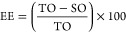
2TO is the total oil content and SO is the surface oil content.

### 2.6 In Vitro Bioaccessibility (Release %) of Encapsulated SCKO

Digestion of encapsulated SCKO was performed under simulated gastrointestinal conditions as previously described in the method of El-Messery et al.^18^ with minor modifications. First, 1.5 mL of the encapsulated oil solution was mixed with 13.5 mL of basal saline (5 mM KCl, 150 mM BHT, and 140 mM NaCl) for 10 min; then, 4.5 mL of simulated gastric fluid (SGF) containing 3.2 g/L pepsin in 1 M HCI was added to this solution at a pH of 2.0 and allowed to react for 1 h. Before adding the simulated intestinal fluid, the pH of the sample was adjusted to 7.5 by adding NaOH (0.1 M). After addition of 4.5 mL of simulated intestinal fluid (SIF) containing 4.76 mg/mL of pancreatin and 5.16 mg/mL of porcine bile extract at a pH of 7.5, the fatty acids were continuously neutralized with NaOH (0.1 M) for 2 h. The fatty acids were then removed from the sample. The experiment was performed in a shaking water bath at 220 rpm, 37 °C. The volume of NaOH added was recorded throughout the digestion. As a result, to calculate the percentage of FFAs released during digestion (%), the following equation^18^ was used:
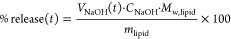
3where *V*_NaOH_(*t*) is the volume of NaOH solution needed to neutralize the FFAs released during the digestion time (*t*). The molarity of the NaOH solution used to titrate the sample is indicated as *C*_NaOH_. *M*_w,lipid_ is the molecular weight of the lipid, while *m*_lipid_ is the total mass of lipid in gram present in the sample during digestion.

### 2.7 Surface Morphology of Dried Coacervates

A Quanta FEG 250 SEM (ThermoFisher Scientific, USA), scanning electron microscopy was used to record the surface structure of the microencapsulated powder at an accelerating voltage of 10.0 kV. The microencapsulated SCKO samples were dispersed on an aluminum pen with an adhesive coating. The pens were coated with a thin gold layer in a Leica vacuum coater at 40 mA for 100 s, using an argon gas purge. The digital photographs were taken at 8000× and 750× magnification.

### 2.8 Statistical Analysis

The findings of the experiments were presented as means with a standard deviation for the three repetitions. The data were statistically analyzed using Minitab 18 (Minitab Ltd., UK). To establish whether or not there was a statistically significant difference between the means, a Duncan multiple range test with a *p*-value of 0.05 was applied.

## 3 Results and Discussion

### 3.1 Effect of Composition of the Wall (Coating) Matrix on the Stability, ζ-Potential, and Particle Size of Coacervates

The coacervate stability, mean droplet diameter (*D*_3,2_), and ζ-potential of coacervates with SCKO prepared with different percentages of core and wall materials are shown in Table 2.

**Table 2:** Physical Characteristics of Complex Coacervates in Terms of Emulsion Stability, Particle Size (nm), and Zeta (ζ-) Potential

sample code	phase separation (%)	particle size (nm)	ζ-potential (mV)
MD 1		185.80 ± 0.4^a^	–36.02 ± 0.5^de^
MD 2		181.72 ± 0.6^a^	–30.40 ± 0.3^bc^
MD 3		178.64 ± 0.1^ab^	–37.04 ± 0.7^e^
MD 4	10 ± 0.1^a^	159.77 ± 1.2^d^	–30.10 ± 0.4^bc^
TH 1		168.55 ± 1.3^c^	–31.16 ± 0.7^bc^
TH 2		173.50 ± 2.3^bc^	–36.13 ± 0.4^de^
TH 3		170.83 ± 2.4^bc^	–28.50 ± 0.6^ab^
TH 4	12 ± 0.2^a^	172.67 ± 1.7^bc^	–26.20 ± 0.1^a^
control-MD:WPC		107.28 ± 1.0^e^	–33.20 ± 0.2^cd^
control-TH:WPC		112.59 ± 0.9^e^	–30.80 ± 0.9^bc^

There was no phase separation in the samples prepared for encapsulation of 1% (w/w) SCKO by different coacervate formulations. However, in the samples with 2% (w/w) SCKO, phase separation was observed in the formulations with 3:1 polysaccharide/protein. This situation was obtained for both MD:WPC (MD4) and TH:WPC samples with a ratio of 3/1 (TH4). The addition of more core material can be attributed to the fact that low wall material favors the coalescence of droplets.^28^ On the other hand, according to Aziz et al.,^29^ the internal structure of the encapsulations can have an impact on their stability. The ratio of the core to wall material and the stirring speed can be changed to adjust the internal structure.^29^ Previously, increasing the polysaccharide concentration in the wall matrix improved the physical stability of the coacervates in dispersion, such that, as seen in Table 2, there was no phase separation in formulations containing 4:1 MD/WPC (MD3) and TH/WPC with 2% (w/w) SCKO (TH3).

The impact of composition of wall material on the ζ-potential of coacervates with different SCKO concentrations is shown in Table 2. All coacervate dispersions had negative ζ-potential and did not significantly affect by the SCKO concentration (see MD1 and MD3; TH1 and TH3), which is a parameter for successful encapsulation.^18^ The negative ζ-potential is caused by the negative charge that the WPC and GA exert at neutral pH. The stability of coacervates with 1 and 2% oil content can be explained to the negative ζ-potential, which may improve the dispersion of the coacervate particles.^30,31^ The ζ-potential of the different coacervate dispersions ranged from −26.20 to −37.04 mV. The ζ-potential of WPC and GA is always negative regardless of pH because the carboxylate groups are the only charged functionalities in the globules.^12^ Chemical or enzymatic cross-linking of the coacervate layer could be a better option to eliminate clumping and creaming of the encapsulated colloids. Covalent cross-linking of WPC and GA makes coacervation irreversible, the pH of the mixture can be changed (above pH 6.0), and repulsion between droplets can occur without destroying the coacervate layer.^32^ The results we obtained for the ζ-potential of coacervates agreed well with the stability values and were consistent with the behavior of WPC:GA complexes in previous studies.^32^ Therefore, it is clear that our results for the ζ-potential (−26.20 to −37.04 mV) of coacervates verify that the surface of the encapsulations is where complex coacervation between WPC and GA occurs.

The mean diameters (*D*_3,2_) of the coacervates with different oil content and wall matrix are listed in Table 2. There were statistically significant differences (*p* < 0.05) for the particle size of capsules as a function of core material concentration. However, the distribution of particle sizes closely resembles a low standard deviation normal distribution. Changes in the core/wall ratio had a considerable impact (*p* < 0.05) on the coacervate particles’ mean diameter (*D*_3,2_), which ranged from 159.77 to 185.80 nm. With the increasing core/wall material ratio, the average droplet size was discovered to rise.^28,30^ In all cases, the coacervates had less than 200 nm of mean diameter, which is likely related to the viscosity of the dispersion. Masters reported that during the atomization process in microfluidization, at a constant atomization rate, the droplet size directly correlated with the viscosity of the dispersion. Higher viscosities resulted in larger droplets during atomization, which produced larger powdered particles.^33^

### 3.2 Results of EE

**Figure 2:**
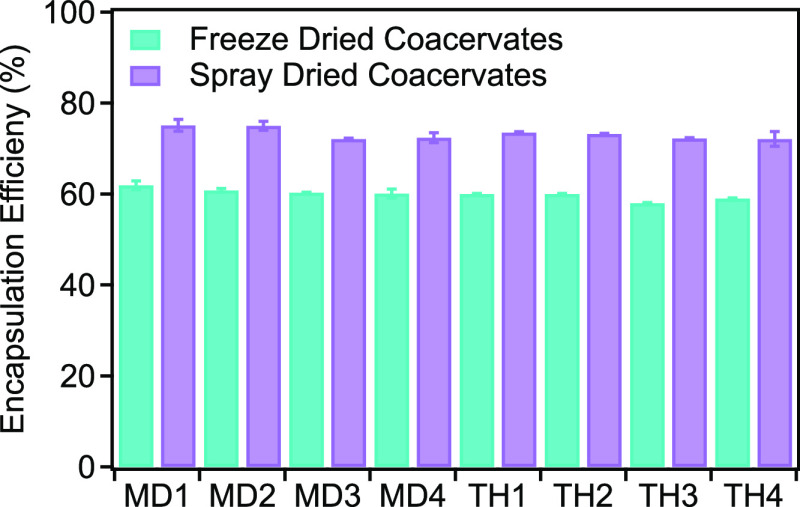
Effect of the wall material concentration and the amount of SCKO in encapsulation formulation on the EE (%) of coacervates.

The EE% indicates the amount of core material retained by the wall material, relative to the total weight of the encapsulants and the total oil used to produce the encapsulants. This efficiency parameter is highly dependent on the concentration of oil used for encapsulation. The ratio of core material to wall, pH, and cross-linking agents are the most important factors affecting EE.^29^Figure 2 shows the EE% of spray-dried and freeze-dried complex coacervates with SCKO.

According to the findings, spray-drying provides a greater EE% than freeze-drying. The surface oil content (unencapsulated oil) of the coacervates did not differ significantly (*p* < 0.05). The samples with 1% (w/w) oil content had higher EE% than samples with 2% (w/w) oil content. The EE% of the encapsulations ranged from 72.10 to 75.10% for the samples prepared by spray-drying and from 58.05 to 61.85% for the freeze-dried samples. However, EE was affected by oil content, so slight differences were observed within the same drying method. In general, small droplets trap oils more efficiently and embed them in the capsule wall matrix, forming more stable structures during the spray-drying process.^28^ Although the poor EE is due to the low gel density at the surface, this is not the only factor but could also be partially caused by unstable dispersion and lead to a large droplet size of oil.^34^

Table 1 demonstrates the impact on EE% of the types and ratio of coating materials as well as the feed’s solid concentration. The rapid formation of a solid surface could be responsible for the lower amount of surface oil because the oil-based core material has less opportunity to leave the surface of the particles.^35^ Some researchers suggest an optimum solid content in the feed.^36^Table 1 reveals that increasing oil concentrations raised the powder’s surface oil content and reduced efficiency for encapsulation. Bertolini et al.^37^ observed a very similar behavior for EE%. This is due to the presence of larger amounts of core materials near the drying surface, which shortens the diffusion path to air/particle contact.^28,37^ Consequently, previous studies^29,38^ reported that the proportion of core material affects the encapsulation efficiency.

### 3.3 Oxidation Stability of SCKO in Spray-Dried and Freeze-Dried Coacervate Dispersions with Different Wall Matrixes

**Figure 3:**
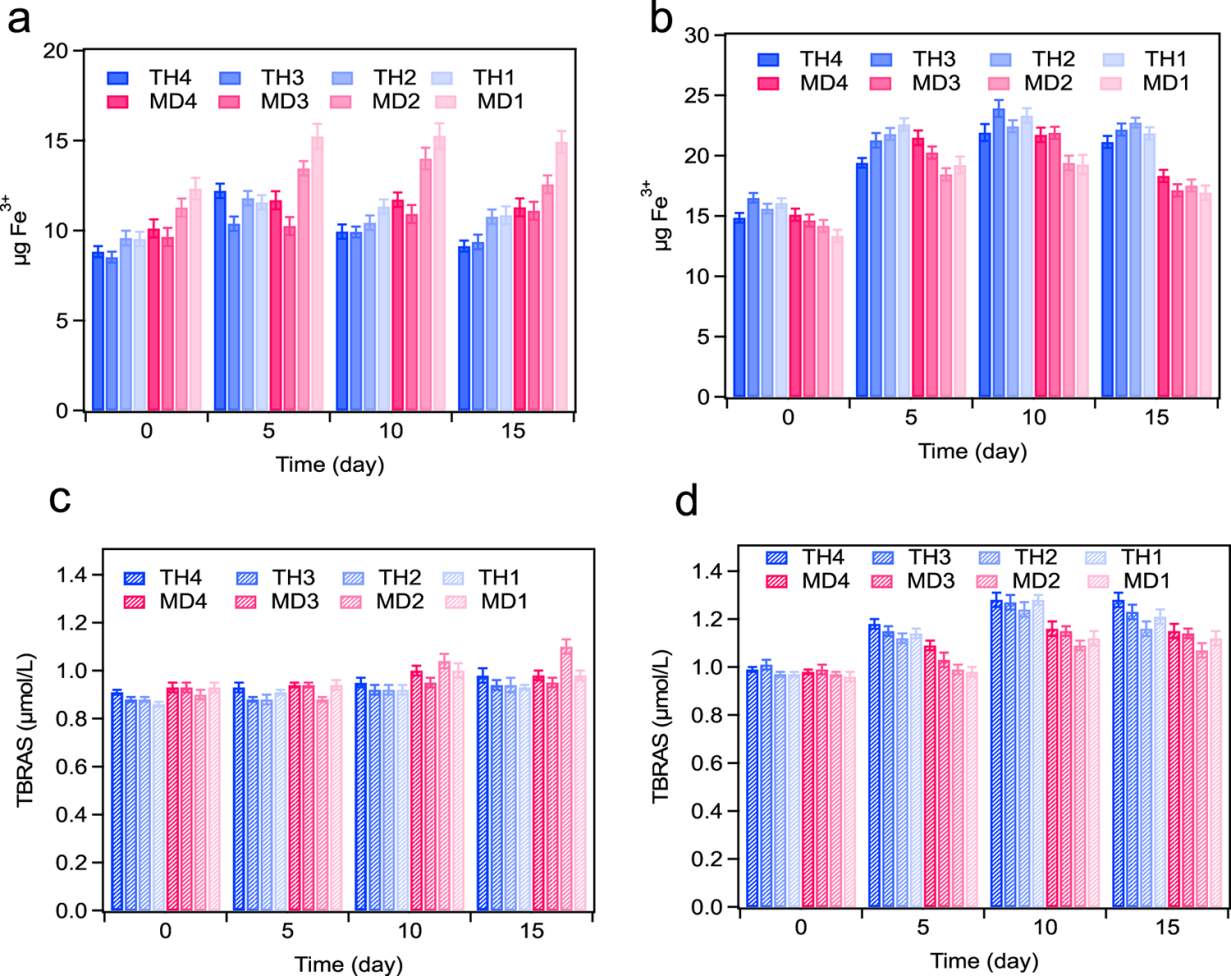
Effect of the drying method (freeze-drying vs spay drying) on oxidative stability of SCKO in different coacervate formulation during at 15 days storage period. A, B changes on the peroxide value, (A) freeze-dried coacervate dispersions; (B) spray-dried coacervate dispersions. C, D changes on the TBRAS value, (C) freeze-dried coacervate dispersions, (D) spray-dried coacervate dispersions. *The formulation of each sample is given in Table 1.

The PV and TBARS method were used to evaluate the oxidative stability of encapsulated SCKO during storage at 55 °C for four weeks. The results of oxidative stability are shown in Figure 3. Yılmaz and Gökmen reported that unencapsulated SCKO oil has lower protection against oxidation with higher formation of hydroperoxides during the first week of storage.^7^

However, when the SCKO was encapsulated by complex coacervates, better protection of oil against oxidation with less hydroperoxide formation was observed even after 2 weeks of storage at 55 °C. The oxidation stability of encapsulated SCKO increased as the percentage of encapsulated oil decreased. During the four weeks of storage, PV increased significantly for all samples (*p* < 0.05). Compared to the other samples, the coacervates with 1% (w/w) oil had significantly lower PV, indicating that they were better protected from oxidation. Initially, all samples had low oxidation levels, ranging from 0.86 to 1.01 μmol/L oil (Figure 3C,D). Spray-dried coacervates constructed from a WPC:TH wall matrix and containing 2% (w/w) SCKO exhibited the highest PV (Figure 3A,B).

### 3.4 *In Vitro* Bioaccessibility of SCKO in Spray- and Freeze-Dried Coacervate Dispersions with Different Wall Matrixes

**Figure 4:**
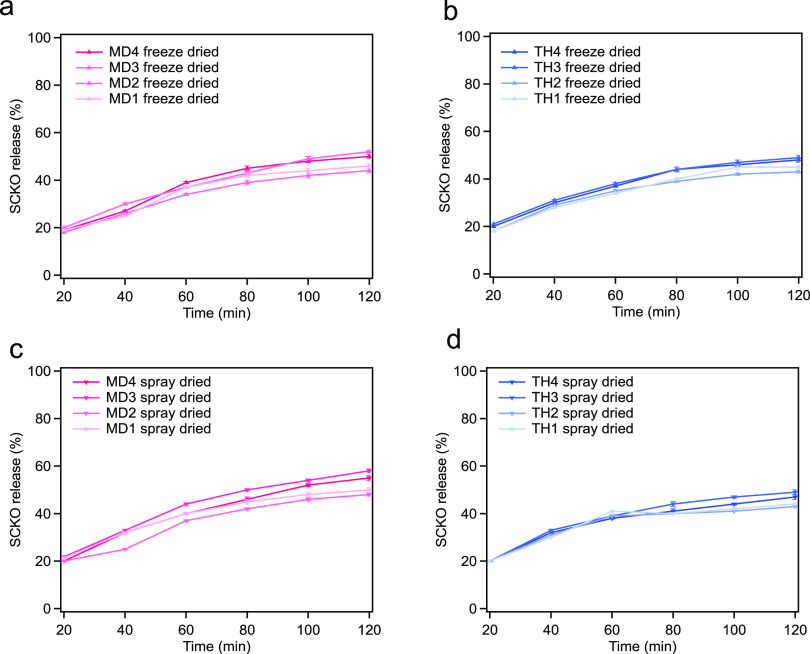
*In vitro* bioaccessibility (%) of SCKO in coacervates with different wall material composition and different ratios during at 2 h in vitro biodegradation. (A, B) Freeze-dried coacervate dispersions with different SCKO concentrations where MD and TH were used as a wall material, respectively; (C, D) spray-dried coacervate dispersions with different SCKO concentrations where MD and TH were used as a wall material, respectively.

Adsorption and accumulation of critical healthy fatty acids are strongly influenced by the release properties of encapsulated oils.^39^ During gastrointestinal digestion controlling the release of bioactive components from the delivery mechanism is critical, the possible advantages of these substances in intracellular delivery will not be achieved otherwise.^20,40^ The release of fatty acids was revealed to be influenced by the percentage of oil contained, the composition of the coacervate wall matrix, and the drying method during *in vitro* digestion of the encapsulated SCKO in a simulated intestinal system. Figure 4 shows *in vitro* bioaccessibility (%) of SCKO in coacervates with different wall material composition and different ratios during at 2 h *in vitro* biodegradation.

The coacervates with low oil content (1%) resulted in lower fatty acid release compared to high oil containing (2%) samples. The release of oil ranged from 18.05 to 52.50% for coacervates prepared by freeze-drying and from 20.05 to 58.04% for microcapsules prepared by spray-drying (Figure 4). According to the results of Chang & Nickerson,^41^ a much larger amount of PUFAs was released during a longer digestion process. Therefore, the omega-3 oil with lower polarity was rarely associated with the wall components, resulting in higher oil release under SGF + SIF conditions, which could be related to the longer digestion process leading to increased degradation of microcapsules by pepsin and pancreatin.

### 3.5 Results of the Surface Morphology

**Figure 5:**
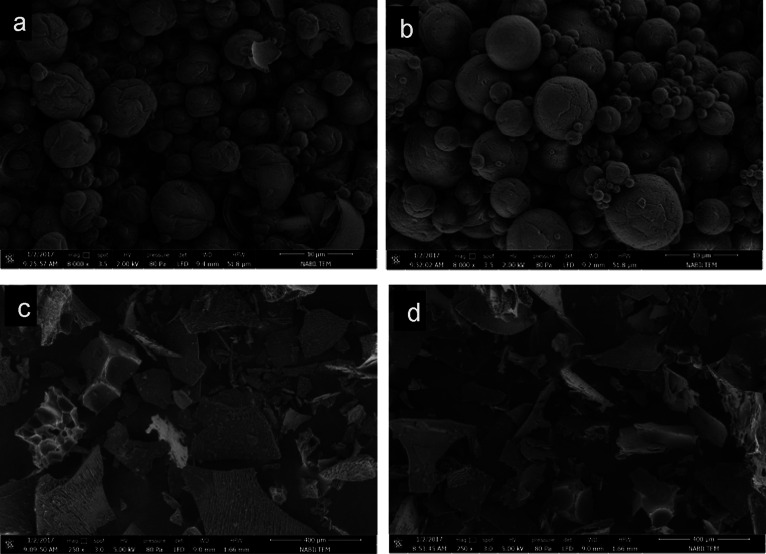
SEM images of dried coacervate dispersions. A, B spray-dried coacervate dispersions, (a) the wall material of coacervates is MD; (b) the wall material of coacervates is TH. C, D freeze-dried coacervate dispersions, (c) wall material of coacervates is MD; and (d) wall material of coacervates is TH.

A powder with low moisture content (less than 5%) was obtained by the freeze-dried and spray-dried coacervates with SCKO. The surface morphology of the dried samples was recorded using SEM. The SEM images are shown in Figure 5.

The capsules, especially the freeze-dried ones, have an irregular shape, whereas the spray-dried particles are primarily spherical in shape with slight surface ridges. The amount of encapsulated oil had no visually significant impact on how the capsules appeared. Spray-dried particles were spherical in shape and varied in size, with visible cracks or fissures. This indicates that the encapsulated particles have lower permeability to gases, which increases the protection and retention of the core material. In addition, variable particle diameters are a common feature of particles produced by spray-drying. Eratte et al. and El-Messey et al. found similar morphological features for spray-dried microcapsules.^12,18^ By sublimating the ice portion of a frozen product, freeze-drying can efficiently dehydrate the studied matrixes. On the other hand, nonuniform and occasionally spongy porous microstructures formed on the freeze-dried matrixes due to the probable formation of voids after sublimation of ice crystals. Zhang et al.^42^ and Silva et al.^43^ observed irregularly shaped particles as microstructures in freeze-dried fish oil microcapsules and Eratte et al. in omega-3 fatty acid encapsulation with SEM.^44^ The irregularly shaped particles were also observed in the microencapsulation of krill oil, by Shi et al.^38^ The spray-dried particles’ external appearance revealed no cracks in the shell. In comparison to freeze-dried microcapsules, the particles’ shell structure was much less porous. In addition, spray-dried microcapsules had significantly higher oxidative stability than freeze-dried microcapsules, partly due to the smaller total surface area and a smaller amount of oil adhered to the surface.^12^

## 4 Conclusions

In this work, one of the food wastes in the food industry, SCKO, was encapsulated and a powder form of complex coacervate was obtained to evaluate it as a functional food ingredient. The effects of wall matrix formulation and different drying techniques on the encapsulation ability of the complex coacervates were investigated in terms of the physical change of the coacervates, the oxidative stability of the encapsulated SCKO during a two-week storage period, and the *in vitro* release (%) of the encapsulated SCKO. The spray-dried coacervates with 1% SCKO offered higher EE and improved oxidative stability during the storage period compared to the freeze-dried ones.

This study also showed that trehalose can be a good alternative to maltodextrin in the preparation of complex coacervates with polysaccharide/protein networks. The coacervates with TH/WPC and MD/WPC wall matrix showed similar structural morphology and physical stability. Moreover, these samples with the same SCKO concentration provided similar protection against oxidative degradation of SCKO during storage, and the bioaccessibility of SCKO in these samples also showed a similar trend. Our results indicate that (i) food processing byproducts have the potential to be a sustainable bioactive source and (ii) dried coacervates composed of a polysaccharide/protein wall matrix provide a suitable platform for the encapsulation of fatty acid sources. Enrichment of foods with encapsulated SCKO to evaluate the physicochemical stability of the encapsulated substances in different food matrixes may be a promising topic for further studies.
